# Parameters setting of Frequency Converter PI regulation to ensure the vacuum of the milking process

**DOI:** 10.1371/journal.pone.0253427

**Published:** 2021-07-01

**Authors:** Jan Kudělka, Jiří Fryč, Vlastimil Slaný, Robert Rouš, Akshaya Raj, Radek Martínek

**Affiliations:** 1 Department of Agricultural, Food and Environmental Engineering, Faculty of AgriSciences, Mendel University in Brno, Brno, Czech Republic; 2 Department of Informatics, Faculty of Business and Economics, Mendel University in Brno, Brno Czech Republic; 3 Department of Cybernetics and Biomedical Engineering, VSB-Technical University Ostrava, FEECS, Poruba, Ostrava, Czech Republic; University of Defence in Belgrade, SERBIA

## Abstract

This paper presents regulation of an asynchronous induction motor so as to create a stable vacuum milk pump using Variable Frequency Drive (VFD). Contribution includes providing information about the usage of the VFD, which regulates the activity of an asynchronous induction motor so that the vacuum pump milking machine creates stable vacuum. The paper describes the functional and time dependence of input values and output parameters of frequency converters at changing electric motor speed. For simulation and verification the milking process a mathematical model of the milking machine was created. The simulation was verified in Matlab/Simulink software. The constructed mathematical model showed symmetric regulation. Control model symmetry was verified at the laboratory of milking machine. The possibility to remove the control valve from milking equipment was proven using the measured data. It was found that constant vacuum values can be maintained. A constant vacuum can be maintained by changing vacuum pump speed. This control is of an accepted standard (ISO 5707: 2007). The power saving control values (on the milking equipment) of the VFD were positive throughout the measuring range. The performance of the milking vacuum pump is normally designed from the maximum air consumption of the milking machine at nominal vacuum (50 kPa), and a performance reserve is added to this. This means that the pump is operated between the ranges 7.53 and 15.06 dm^3^ s^−1^. By using a vacuum pump controlled by a VFD, power savings can be achieved from 32.50% to 54.02% compared to a control valve.

## Introduction

During machine milking of dairy cows, the milk is acquired by vacuum. The vacuum beneath the teat (claw vacuum) changes during the course of milking [1]. The basic element of the milking machine is a vacuum pump. It is crucial to ensure stable vacuum to achieve required performance of the milking equipment. The vacuum pump provides the necessary vacuum in a milking system [2, 3]. Milking machines currently use rotary vacuum pumps [4]. Vacuum pump sets driven by an asynchronous motor with short armature must meet operational requirements (milking, cleaning, and sanitation). Operational requirements are still or intermittent [5, 6]. Vacuum in the pipe lines (40–50 kPa) and the theoretical performance of the vacuum pump are given by an international technical standard ISO 5707: 2007 [7]. Milking machine settings affect both milking characteristics and teat tissue condition [8–10]. Low vacuum inside the liner may reduce liner closure and result in less pressure acting on the teat during the massage phase. Additionally, if the transport of milk from the teat through the short milk tube is based on a low vacuum, the vacuum can reverse at the moment the liner opens the a-phase of pulsation, potentially causing a reflux of milk back from the claw to the teat tip and into the udder [11, 12]. This may increase the risk of cross-infections between quarters. The vacuum control system is based on a balance between the partial vacuum produced inside the milking machine resisting force of a weight of control valve [13]. Weighted regulators use a ‘dead weight’ or a weight mounted on a lever system. The force weight of operating to close the control valve is the same as the force of the system vacuum operating to open it. Spring actuated regulators use the same control principle. Only closing force on the control valve is supplied by a spring. The vacuum pump works at a constant speed. For all scenarios, the use of a vacuum pump at the maximum expected capacity of the amount of air taken remains. The control valve opens if more air is removed than allowed, to increase the amount of air allowed through the controller. The asynchronous motor and vacuum pump work at full power. Owning a milking process consumes a disproportionate amount of electricity. From a technical point of view it is necessary to ensure a stable vacuum in the system with the optimum operating parameters of the asynchronous motor. The optimum operating parameters of the asynchronous motor are speed of engine, torque and power consumption. For this purpose, it is possible to use the principles of Variable Frequency Drive (VFD), which allows the speed to be adjusted to the required values. VFD controllers are a new way of controlling vacuum pump of milking machine. VFD adjusts the amount of air taken from the milking machine, instead of adjusting the amount of air supplied to the milking machine [14]. Properly set VDF provides comparable control of vacuum values with conventional regulators [15, 16]. VDF system reduces electricity consumption by pumping the current air-flow rate into the system at optimum operating parameters of the electric motor [17–19]. Energy consumption is reduced upto 30 to 50% compared to constant vacuum pump speed [16, 20]. Current of large electric motors is reduced VFD controllers. This can be an important advantage as operated on some rural power distribution systems [17]. When using VDF, the magnitude of the momentary motor starting neutral voltages is reduced. The aim of the paper is to determine and correctly set the determinant parameters of the VDF so as to maintain the vacuum stability in the system at the minimum power input of the electric motor. The consumption of electric energy by an electric motor controlled by a frequency converter is recorded and evaluated. The created mathematical model in Matlab/ Simulink 2014a will verify whether it is a symmetric or asymmetric control system. Setting of the PI controller on a real device based on a symmetric/ asymmetric model guarantees of a stable vacuum value. Proper setting of parameters the VDF will save electricity during the milking process. The work presents the design of an artificial cognitive control system. They are based on the shared circuit model. The principle of shared circuits model (SCM) includes: Control, mirroring, and simulation. SCM can enable imitation, deliberation, and mindreading [21]. Neurofuzzy systems, the semantic transparency neurofuzzy systems have certain advantages that allow us to combine them all, and the intrinsic robust nature of fuzzy systems can be applied. [22, 23].

## Materials and methods

**Fig 1:**
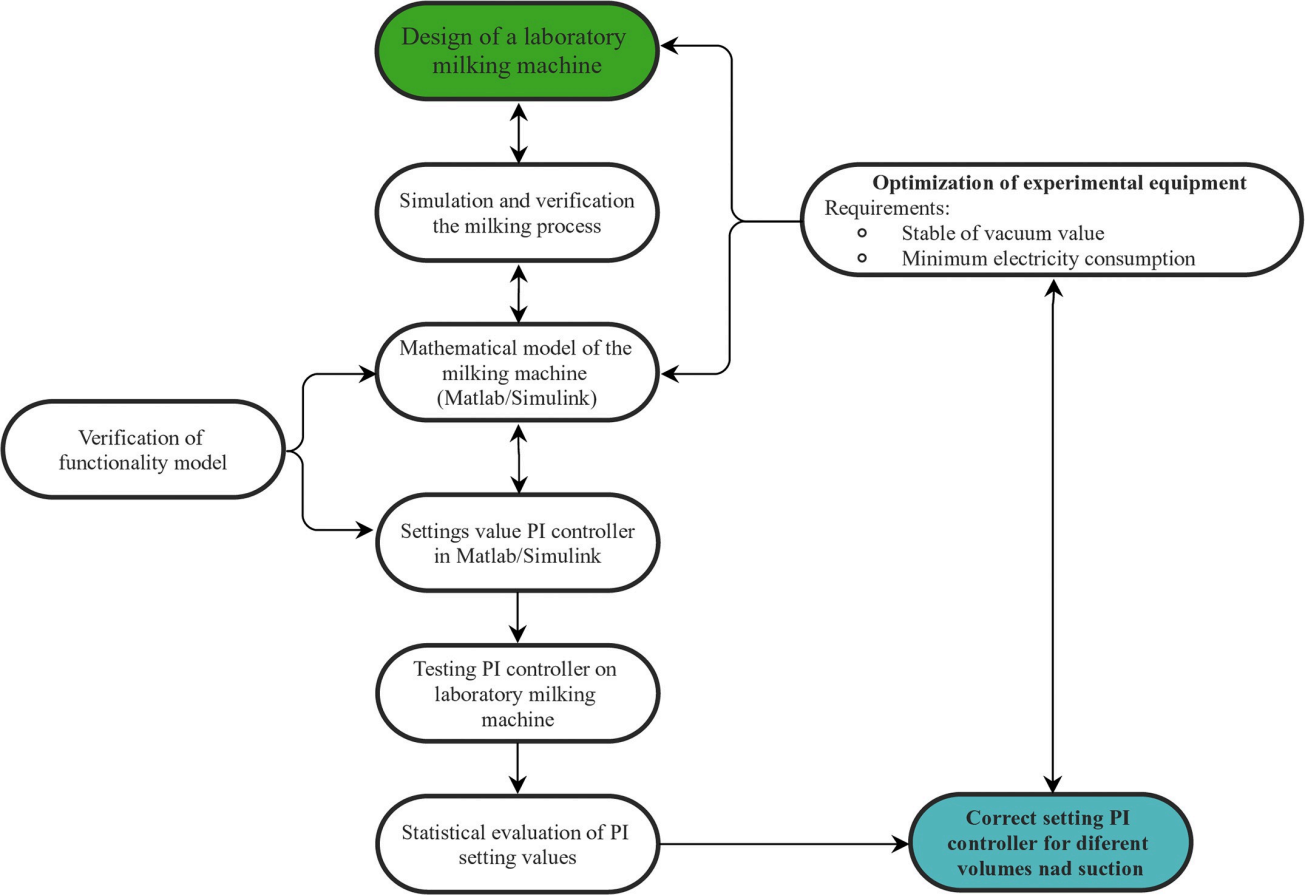
Block diagram of setting and testing parameters of Frequency Converter PI.

The entire process of Setting and testing parameters of Frequency Converter PI is described in Fig 1.

**Fig 2:**
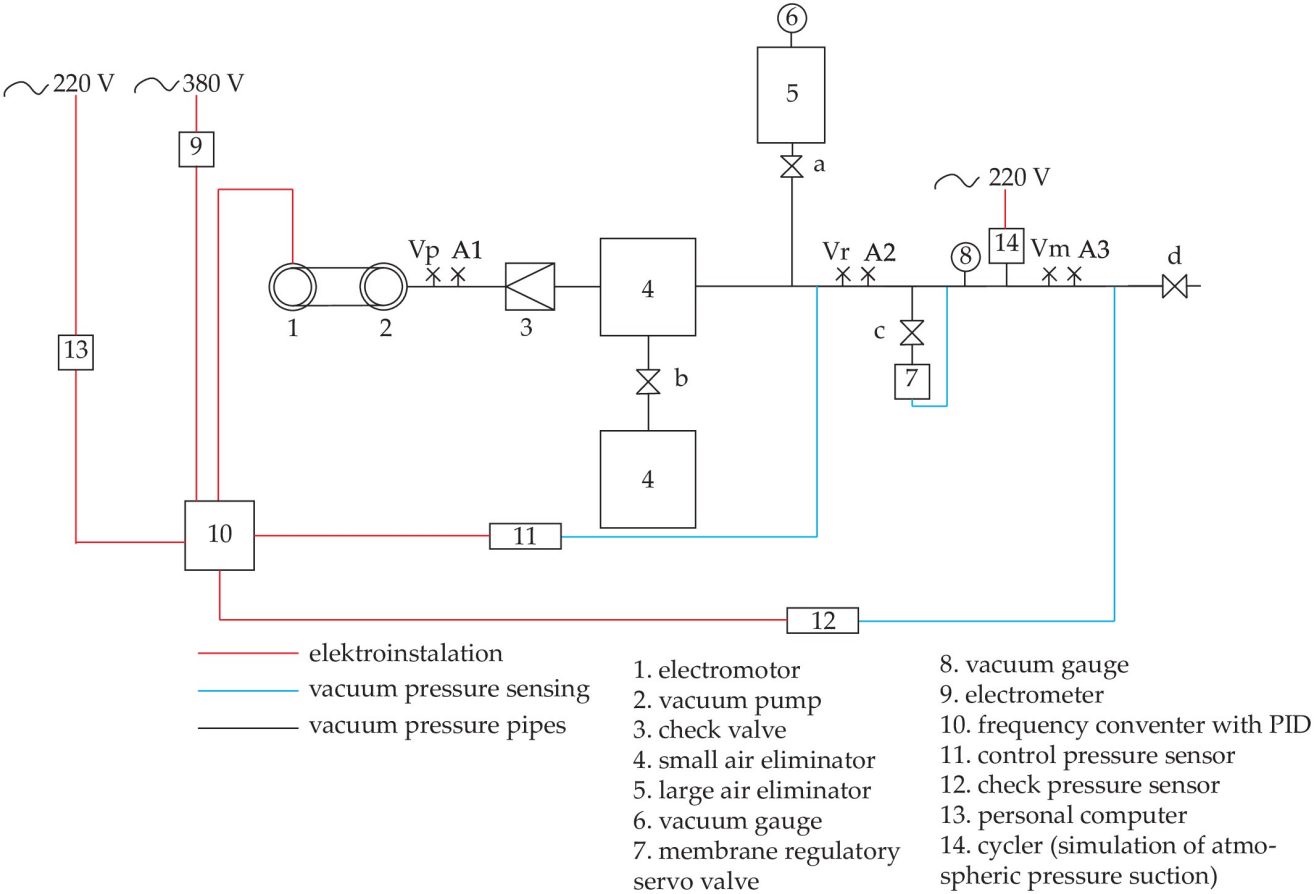
Scheme of the reference milking equipment.

To accomplish the objective an experimental milking equipment was constructed to simulate and verify the milking process. The milking equipment is described schematically in Fig 2. The assembled system consists of a rotary blading vacuum pump (SACCO1600) with a theoretical performance of 26.5 dm^3^ s^−1^, a three-phase asynchronous electric motor SIEMENS with a theoretical output of 4.1 kW and frequency converter Siemens SINAMICS G120 with a control unit (Siemens CU230P-2). The milking equipment was designed to comply with International Standard ISO 5707: 2007 [7]. To test the functionality of the milking equipment, mechanical tests were performed according to the International Standard ISO 6690: 2007 [24].

A rotary vacuum pump, asynchronous electric motor SIEMENS, frequency converter Siemens SINAMICS G120 with a control unit (Siemens CU230P-2), cycler and Siemens Starter GUi were used for the experiment. For regulation, several nonlinear controls can be used to stabilize the chaotic systems. It is an approach to sliding mode control and a combination with robust adaptive features [25]. Adaptive fuzzy slide mode controllers for chaotic systems can be applied [26–28]. The application can use the idea of logic control of chaotic systems proposed of the Rikitake system on the basis of Takagi—Sugeno—Kang (TSK) fuzzy logic controllers (FLCs) [29, 30]. When controlling the electric motor using the control unit Siemens SINAMICS G120P BT, prior knowledge of the input parameters is necessary. These parameters affect the stability of vacuum during milking. Laboratory milking equipment were identified and modeled for this reason. To create a system model and simulate it for different controller settings with different characteristics, the following steps were performed:

1. Experimental measurement of transition characteristics for selected volumes.
2. Identification of systems by approximation.
3. Systems creation in Matlab / Simulink 2014a.
4. Model verification based on measured data.

The overall dynamics of the system behavior was measured as a transient characteristic (response to unit jump). Each selected volume (V1 = 10.1639 m^3^, V2 = 0.2895 m^3^, V3 = 0.3739 m^3^, V4 = 0.4995 m^3^) was considered as a separate system. The same procedure was used for various suction values (Qsv = 0 m^3^ s^−1^, 5 m^3^ s^−1^, 10 m^3^ s^−1^, 13,3 m^3^ s^−1^). Transient characteristics were measured by recording the increasing vacuum in the system. The identification of the systems was performed by approximation of the transient characteristics by the general transfer function prescription (1).

$$\begin{array}{c} G\left(s\right)=\frac{k}{\left(Ts+1\right)} \end{array}$$ (1)

*k* is amplification of the first order system

*Ts* is time constant of inertia

**Fig 3:**
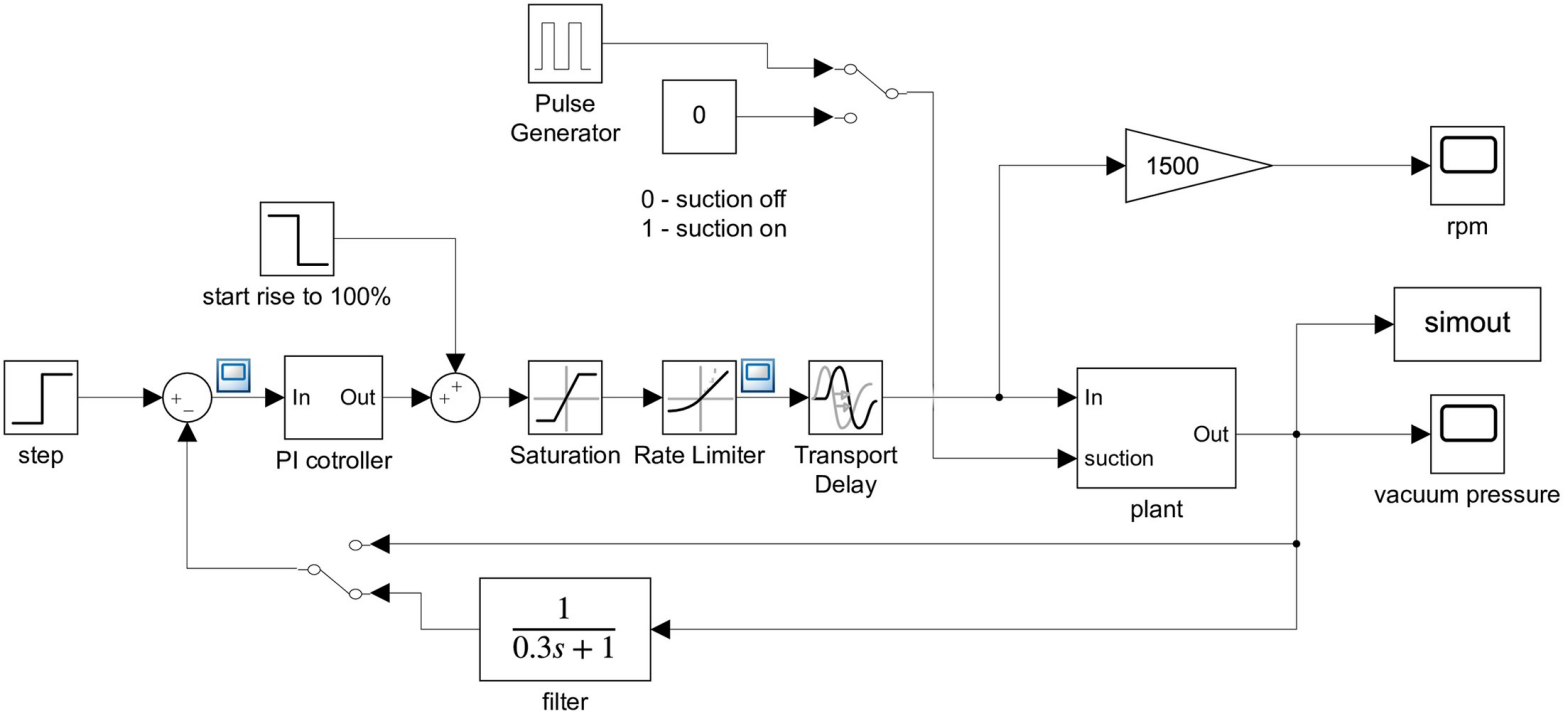
System model in Matlab / Simulink 2014a.

This kind of system was chosen because of the physical nature of the measured system as it is a simple energy storage system. The use of a first order system is also a commonly used method for identifying higher order systems if their course is non-oscillatory [31, 32]. On approximation, two parameters were changed based on Eq (1). Approximated mathematical formulas for individual measured transient characteristics were created by approximately setting the parameters. The control model was created in Matlab / Simulink 2014a. A simple regulator control loop was used due to the chosen control method. Experimental and empirical setting methods were chosen to verify the model. When creating the system model, the settings of the frequency converter and the differences from the basic control scheme were taken into account. The methods were characterized by setting the parameters of the controller connected to the actual controlled system. Ziegler-Nichols methods are classic representatives of the experimental setting. The Ziegler-Nichols method of critical parameters was used to set the individual PI constants. [33, 34]. The frequency inverter settings were taken into account, when creating the system models. Each model in Matlab/ Simulink 2014a created represented one volume and one specific constant suction (Fig 3).

Each Model consisted of the following blocks:

- Step—The block represents the required vacuum value in the system.
- PI controller—The block represents the controller and its settings for the given simulation.
- Saturation—The block represents the limitation of the maximum action of the controller. Relative to the real system—the motor driving the pump has a maximum achievable speed.
- Rate Limiter—The block represents the maximum possible change in the speed of action. That is, the maximum possible change in the speed of the pump. This setting corresponds to the setting of the start and stop limits in the frequency converter.
- Transport Delay—The block represents a transport delay. The pump is not able to react immediately to changes in motor speed.
- Plant—Regulated system—This block is consisted of transfer functions for the set constant suction and the suction added by the cycler.
- Filter—The block represents a filter that is set in the frequency converter for filtering the controlled variable.
- Simout—The block is used to save the simulation results in the Matlab environment for possible further work.
- The pulse generator—Block is a timer with a switching period set as in a real experiment (34 s).
- Vacuum and speed—The blocks are displays of simulated speed and vacuum values in the system.

**Fig 4:**
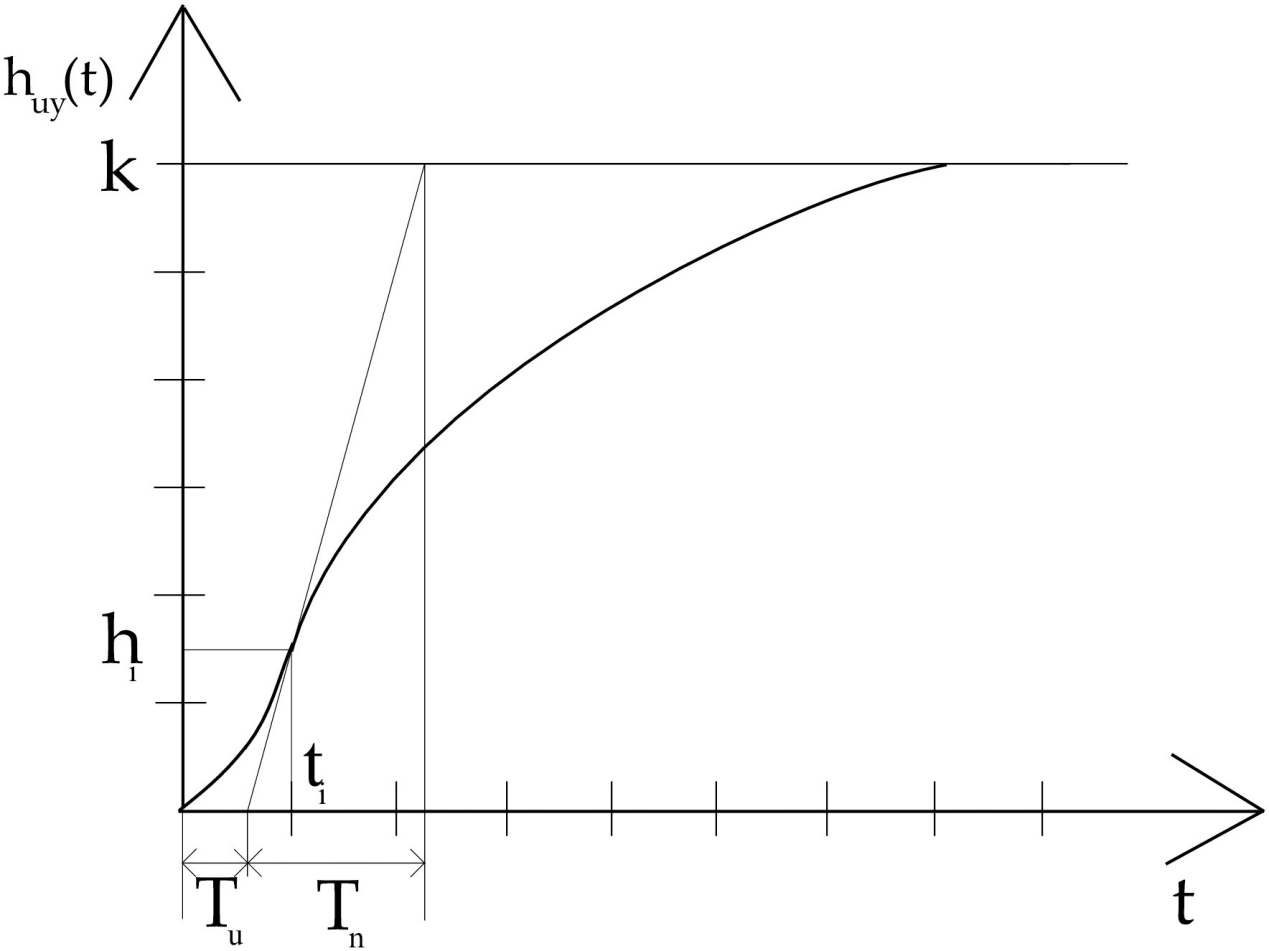
The transient characteristic of the proportional non-oscillating controlled system.

Assembled models are closer to a real system than theoretical concepts. Each created model represents one volume and one specific continuous air flow. Parameters of Frequency Converter were set using Ziegler-Nichols’ Critical Parameter Method. The verification was performed using the Ziegler -Nichols method of the transition characteristic. The transition characteristic method is also called the open control circuit method. It is based on the transient characteristic of the proportional non-oscillating controlled system, the course of which is shown in Fig 4. From the course of the transition characteristic, the delay time Tu, and the rise time Tn are determined.

Values of adjustable parameters of analog PID controllers Ziegler -Nichols transition characteristic method are in Table 1.

**Table 1:** PID controllers Zigler-Nichols transition characteristic method.

Regulator type	Kp	T_I_	T_D_
P	$\frac{T_{n}}{k\cdot T_{u}}$	–	–
PI	$0.9\cdot \frac{T_{n}}{k\cdot T_{u}}$	3.33 ⋅ *T*_*u*_	–
PID	$1.2\cdot \frac{T_{n}}{k\cdot T_{u}}$	2 ⋅ *T*_*u*_	0.5 ⋅ *T*_*u*_

The individual constants are explained in Fig 4. Correct settings were measured on a reference milking equipment, and later the measuremnets were compared with the simulation. Laboratory measurements were designed to simulate the characteristic condition of the milking process on farms. The purpose is to verify the mathematical models compiled in Matlab / Simulink 2014a. Setting of Frequency Converter should ensure correct control of the drive while maintaining a stable negative pressure and varying atmospheric air flow and vacuum system volume. Measurements are performed as follows: The vacuum pump is activated, the vacuum pump works at maximum speed (1340 rpm) and is activated (42 kPa) for fifteen minutes. The pressure is controlled by the control valve. In the control unit of Siemens SINAMICS G120P BT is set a nominal vacuum value (42 kPa) by the program Starter Commissioning. The current vacuum (42 kPa) matches the output of the current 13.28 mA sensor. Checking valves are set for individual volume sizes (V1 = 0.1639 m^3^—V4 = 0.4995 m^3^). The SAC FLOWING METER is connected to the vacuum system according to Fig 1. The flow meter is set to 10 dm^3^ s^−1^ and opened gradually to 15 dm^3^ s^−1^. The flow rate and vacuum regulation are limited by the minimum and maximum speed of the electric motor (750 to 1450 rpm). For simulation of the “fault condition”, a “timer” is connected to the vacuum pipe. The timer sets atmospheric air every 34 seconds into the pipeline in volume *Q*_*svp*_ = 1.72 dm^3^ s^−1^. The value of vacuum stability is measured and recorded. The value of vacuum in the milking machine is recorded using the STARTER Commissioning TRACE software. Control PI must correctly set the determinant parameters of the VDF so as to maintain the vacuum stability in the system at the minimum power input of the electric motor. Each selected volume (V1 = 0.1639 m^3^, V2 = 0.2895 m^3^, V3 = 0.3739 m^3^, V4 = 0.4995 m^3^) is considered as a separate system. The same procedure is used for various suction values (*Q*_*sv*_ = 0 dm^3^ s^−1^, 5 dm^3^ s^−1^, 10 dm^3^ s^−1^, 13.3 dm^3^ s^−1^). Constant vacuum values will be maintained by changing the speed of the vacuum pump with a correct converter control. This control is accepted standard (ISO 5707: 2007). The parameters of proportional amplification (KP) and integration time (TI) are matched to the values of the assembled mathematical model. The measured values of the output current sensed by the BD-SENSORS DMP 3311 current sensor are written by the program as a percentage. Therefore, they are converted to the actual vacuum ps by the means of a similarity relationship (2).

$$\begin{array}{c} P_{s}=\frac{20-I_{p}}{100}\cdot \%I_{s}\cdot 6.25\;\left[\text{kPa}\right] \end{array}$$ (2)

*I*_*p*_ is the required electric current set at the vacuum sensor [mA]

%*I*_*s*_ is the actual percentage of the current sensed by the current sensor [%]

Measured values of vacuum under control with PI control were statistically evaluated and compared with classical regulation. The one-sample that was tested determined whether the sample diameter differs from the specified value of μ0 (target value 42 kPa) with the availability of sample variance *s*^2^ [35]. The arithmetic mean and variance of the sample are calculated, then the test criterion t according to (3).

$$\begin{array}{c} t=\frac{\bar{x}-\mu }{\sqrt{\frac{s^{2}}{n}}} \end{array}$$ (3)

$\bar{x}$ is sample average

*μ* is mean value of the population

*n* is number of members of the sample

Two-sample F-test according to [36] tested equality of variance. As a result of a p value of less than 0.05 (p < 0.05) at all observed settings, it is evident that the groups have different variance. Therefore, a two-sample t-test with dispersion inequality is used to test the mean values of two independent selections (control settings). The measured values (*μ*1) of the vacuum for all volumes (V1 to V4) and of the air suction (*Q*_*sv*1_ to *Q*_*sv*2_) is the data measured during the regulation with the help of a control valve. The hypothesis of equality of mean values *μ*1 and *μ*2 is tested. The test criterion is calculated according to formula (4).

$$\begin{array}{c} t=\frac{{\bar{x}}_{1}-{\bar{x}}_{2}}{\sqrt{\frac{s_{1}^{2}}{n_{1}}}+\sqrt{\frac{s_{2}^{2}}{n_{2}}}} \end{array}$$ (4)

$\bar{x}$ is sample average

*n* is number of members of the sample

$s_{1,2}^{2}$ is sample variance

The hypothesis *H*_0_ at the significance level *α* = 0.05 on the similarity of mean values is rejected if the value t exceeds the critical limit. When setting the PI controller parameters, a statistically inconclusive difference with the control valve was confirmed by two-sample t-test with dispersion unevenness (the null hypothesis *H*_0_ was confirmed), the evaluation was performed using basic descriptive statistics.

## Results

**Fig 5:**
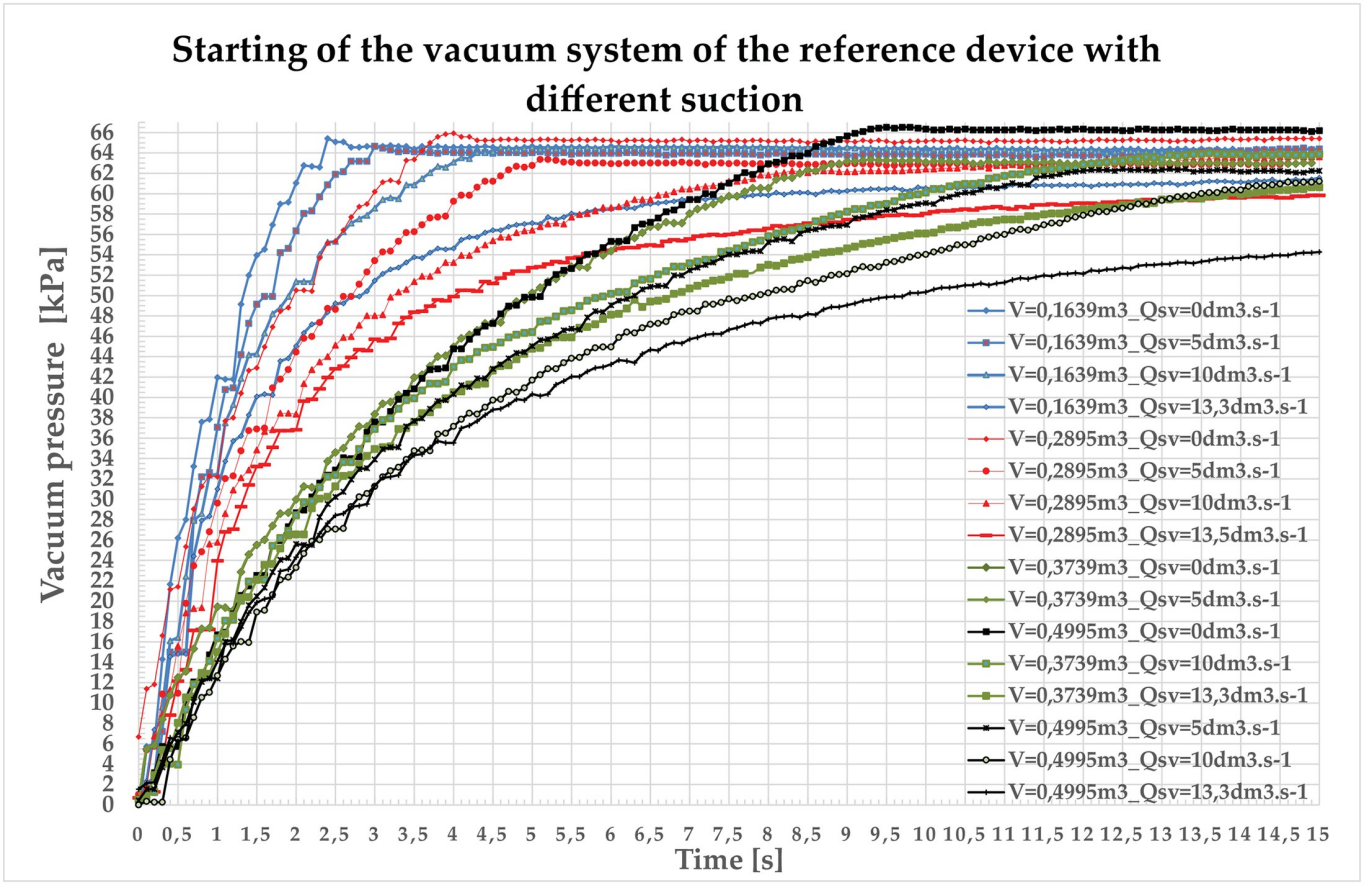
Starting of the vacuum system of the reference device with different suction.

On the assembled milking equipment the transient characteristic is measured (V1 = 0.1639 m^3^—V4 = 0.4995 m^3^) defined suction (0–13.3 dm^3^ s^−1^). This can be observed in Fig 5. The Frequency Converter with PI controller was deactivated, the asynchronous induction motor of vacuum pump was not regulated, the vacuum pump rotated at a maximum speed of 1340 rpm. Transition characteristics are defined as system time responses per unit step at zero initial conditions. First-order characteristics without time delay has been approximated with sufficient precision [37].

Approximate transition functions for individual volumes and different pickings are described in Table 2.

**Table 2:** Volumes of approximate functions and times constants.

Volume	0 dm^3^ s^−1^	5 dm^3^ s^−1^	10 dm^3^ s^−1^	13,3 dm^3^ s^−1^
0,1639 m^3^	$G_{s}=\frac{76}{1,25s+1}$	$G_{s}=\frac{69}{1,25s+1}$	$G_{s}=\frac{64,5}{1,25s+1}$	$G_{s}=\frac{61,5}{1,65s+1}$
0,2895 m^3^	$G_{s}=\frac{76}{1,98s+1}$	$G_{s}=\frac{69}{1,98s+1}$	$G_{s}=\frac{64,5}{1,98s+1}$	$G_{s}=\frac{61,5}{2,1s+1}$
0,3739 m^3^	$G_{s}=\frac{76}{3,8s+1}$	$G_{s}=\frac{69}{3,8s+1}$	$G_{s}=\frac{64,5}{3,8s+1}$	$G_{s}=\frac{61,5}{3,8s+1}$
0,4995 m^3^	$G_{s}=\frac{76}{4,7s+1}$	$G_{s}=\frac{69}{4,9s+1}$	$G_{s}=\frac{64,5}{4,7s+1}$	$G_{s}=\frac{61,5}{4,3s+1}$

**Fig 6:**
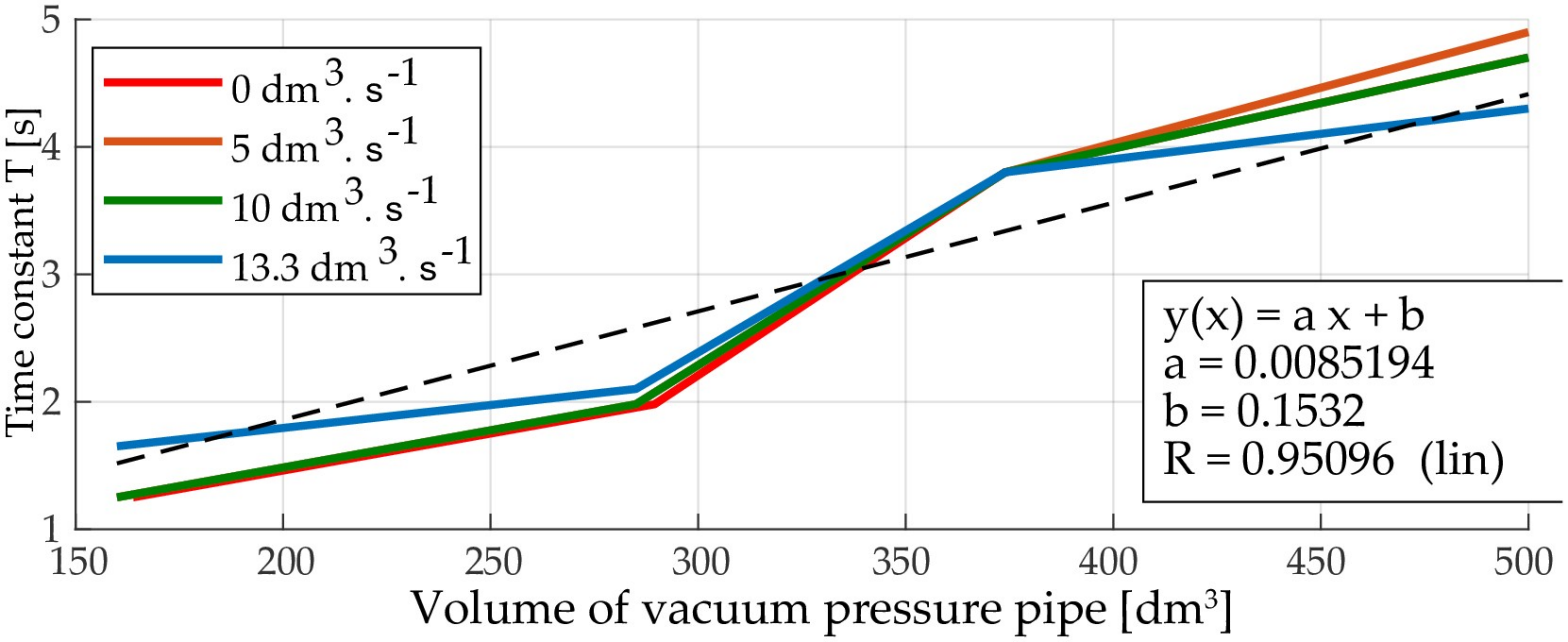
Dependence of time constant on volume.

The results show that the volume of the vacuum pipe affects only the time constant of the system. The maximum available vacuum is the same. The time constant of the system increases with the increasing volume of vacuum pipe (Fig 6).

For variable volumes (V1 = 1639 m^3^ to V4 = 0.4995 m^3^), vacuum values are set, measured and recorded. The setting of amplifying components (P) and integrating time (I) on the real system are chosen based on simulations compiled in the Matlab / Simulink 2014a program. The real PI controller is part of the VFD SINAMICS G120. The parameters settings are made in the computer control unit with the Starter GUi software. The settings of the frequency converter SINAMICS G120 were taken into account when creating the system model and the differences from the model basic control scheme. This brings the model closer to the real system than to the theoretical concept. In addition to the PI values, the following can be set using Starter software, for example:

- The required vacuum value in the system.
- Limitation of the maximum action of the controller.
- The maximum possible change in the speed of the action.
- Transport Delay.
- Transfer functions for the set constant suction and the suction added by the cycler.

**Fig 7:**
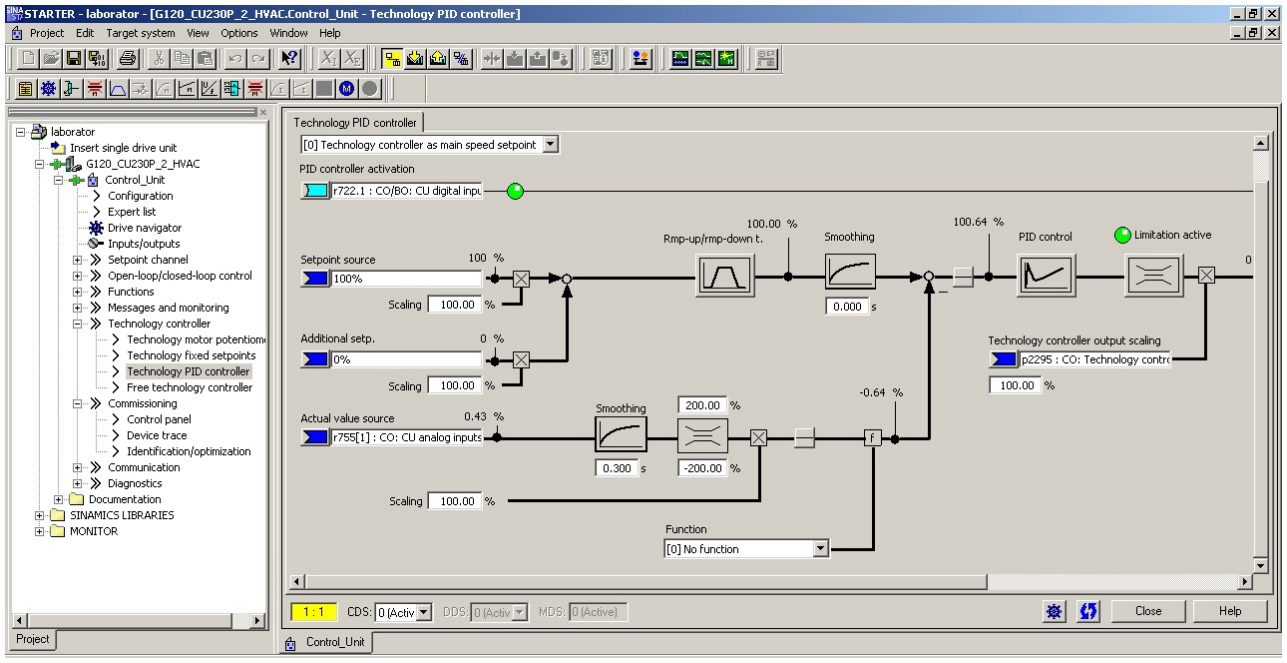
Work desktop of PI controller the software Starter.

In the Starter software the adjustable parameters, the output values of the frequency converter and the electric motor can be monitored. Each created model represents one volume and one specific constant suction. These parameters were represented in the approximated system and its transfer function. On the PI controller, it is possible to select different parameters of the amplifier (P) and the integration time (I). The software desktop is shown in the Fig 7.

**Fig 8:**
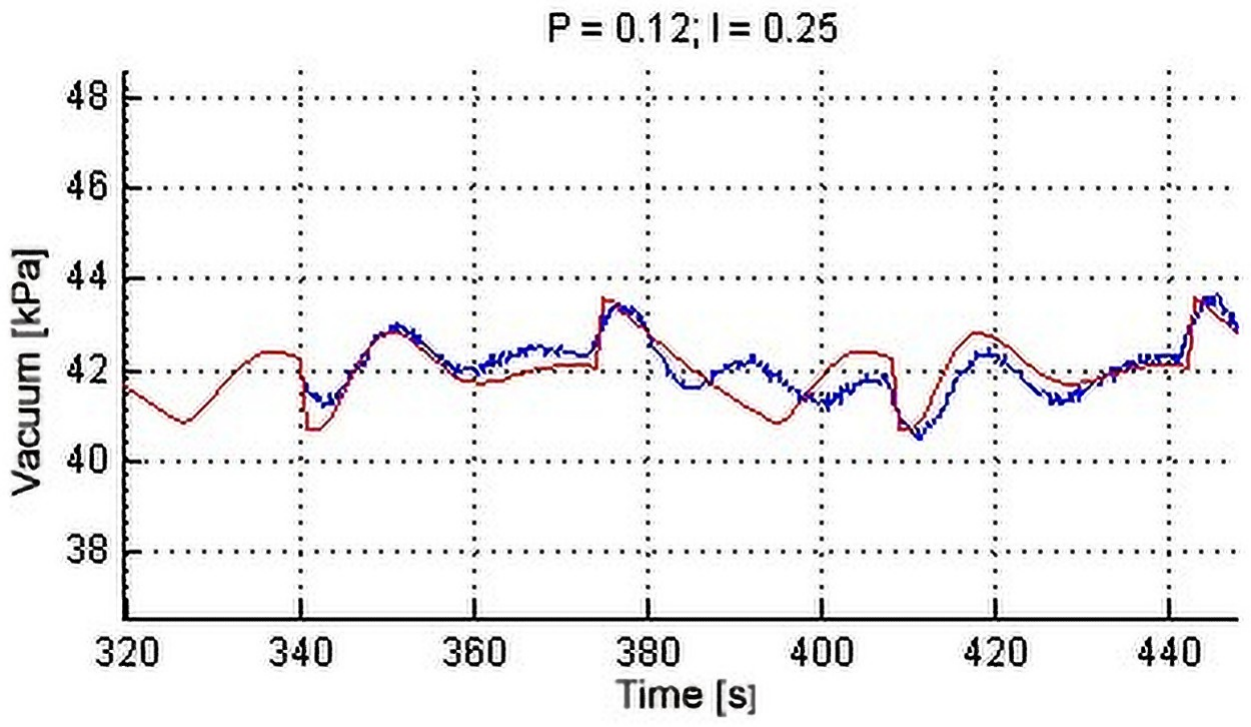
Comparison of simulation with real system (optimal setting).

Process control systems (SCM) enable more efficient and faster control. The benefit of a modified system is the organization and optimization of behavior. The assembled model is an intelligent system-an imitation of the self-human mind evolution [33]. Simulation values of vacuum stability for the selected suction, air volume and setting of PI controller constants were performed on the assembled model. The regulator was set on the basis of determining the maximum achievable value vacuum. The Ziegler-Nichols method of critical parameters was used to set the individual PI constants. Fig 8 shows a comparison of the simulation (Matlab / Simulink 2014a) and the real milking system for the volume of 0.4995 m^3^ when sucking 10 + 1.72 dm^3^ s^−1^. It is seen here that the model copies the behavior of the real system.

Limiting factors for parameter setting (PI) of the electric motor SIEMENS were the actual pump output (25.1 dm^3^ s^−1^), the maximum speed (1450 rpm) and the minimum speed (718 rpm). The parameter setting (PI) when controlling the frequency converter was carried out with air suction between 10 and 15 dm^3^ s^−1^ by regular cyclic loading of 1.72 dm^3^ s^−1^ and period of 34 s. From the functional “optimal” PI control, we derive the dependency of the proportional component (P) and the integration time (I) [38].

**Fig 9:**
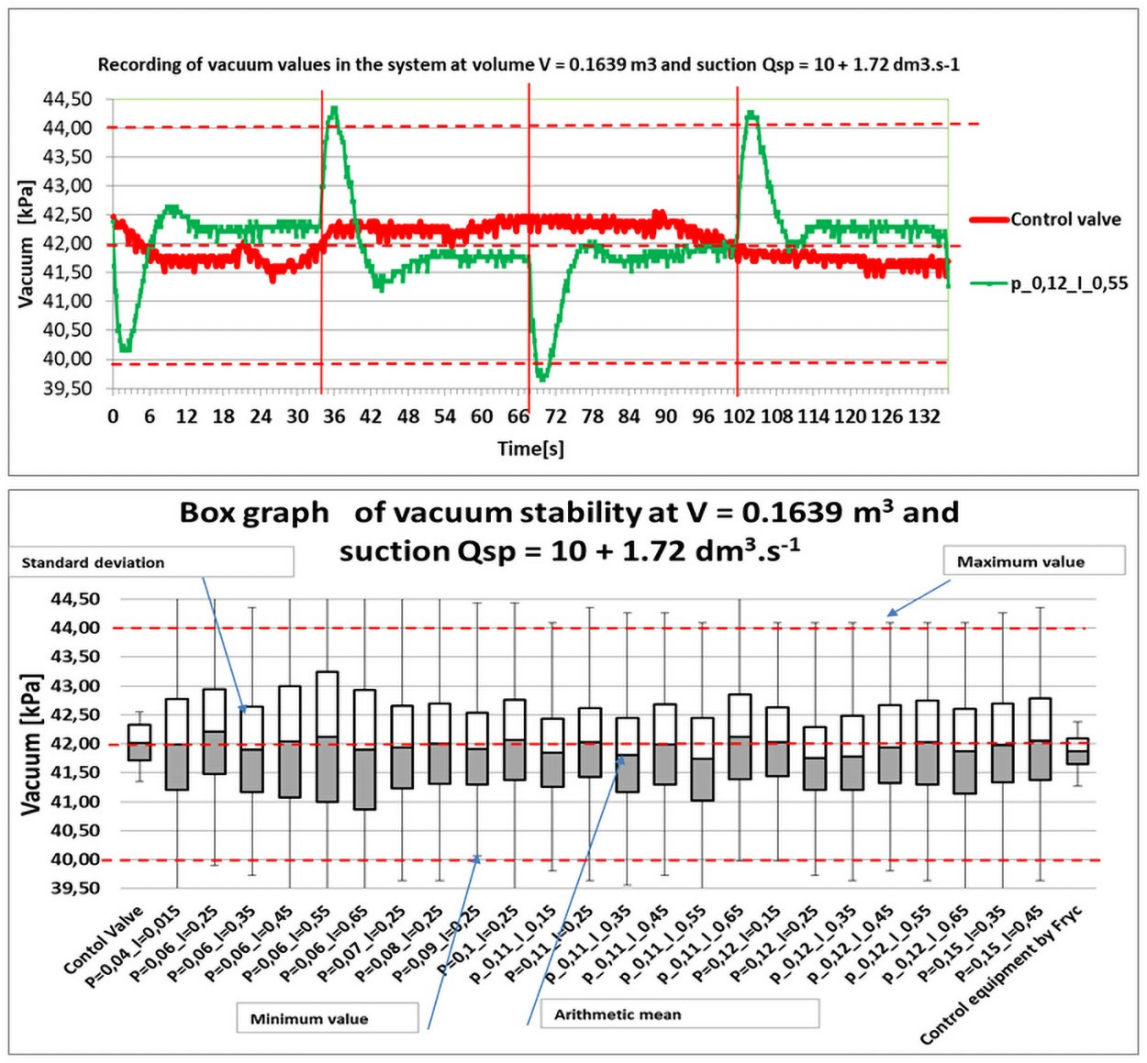
Graphical representations of vacuum stability.

Non-oscillating setting from simulations were selected for these suction variants: 10 + 1.72 dm^3^ s^−1^ to 15 + 1.72 dm^3^ s^−1^. A single-sample t-test of the sample diameter (individual PI settings) and the diameter of the base set (setpoints 42 kPa) was performed on the measured vacuum values. As a result of the large sample size, the t-test eliminated the effect of the variance of values and resulted as statistically inconclusive in all tested settings with an arithmetic mean (approaching 42 kPa) regardless of the variance of sampling. The test was evaluated at a significant level of *α* = 0.05. For periodically non-oscillating settings, simulated on a mathematical model and measured in a real system, two-sample t-tests were performed with uneven variance. The results of these statistics are clearly described in Table 3. For clarity is given only in suction of 10 + 1.72 dm^3^ s^−1^. The verification of the accuracy of the test was done by evaluating the basic descriptive statistics at a 95% confidence level. Graphical representations of vacuum stability courses were supplemented by illustrative box graphs in Fig 9.

**Table 3:** The accuracy of the test was verified using basic descriptive statistics.

Basic descriptive statistics: *V* = 0.1639 m^3^, *Qsp* = 10 + 1, 72dm^3^ s^−1^					
	Control valve	Control device design by Fryc	P = 0,04;I = 0,15	P = 0,06;I = 0,45	P = 0,08;I = 0,25
Arithmetic mean [kPa]	42,02	41,87	42,00	42,06	42,01
Median [kPa]	42,04	41,78	41,95	42,12	41,95
Mode [kPa]	42,29	41,78	42,29	42,29	42,29
Minimum value [kPa]	41,35	41,27	39,30	39,30	39,64
Maximum value [kPa]	42,55	42,38	44,61	44,86	44,69
Standard deviation [kPa]	0,3	0,22	0,78	0,96	0.7
Variation range [kPa]	1,2	1,11	5,31	5,57	5,05
Variance of selection[-]	0,09	0,05	0,61	0,92	0,49
	P = 0,11;I = 0,45	P = 0,12;I = 0,15	P = 0,12;I = 0,55	P = 0,15;I = 0,45	
Arithmetic mean [kPa]	41,99	42,03	42,02	42,05	
Median [kPa]	41,95	42,12	41,95	42,12	
Mode [kPa]	41,78	42,29	42,29	42,29	
Minimum value [kPa]	39,73	39,98	39,64	39,64	
Maximum value [kPa]	44,26	44,09	44,35	44,57	
Standard deviation [kPa]	0,69	0,96	0,73	0,74	
Variation range [kPa]	4,54	4,11	4,71	4,93	
Variance of selection[-]	0,47	0,92	0,53	0,46	

**Fig 10:**
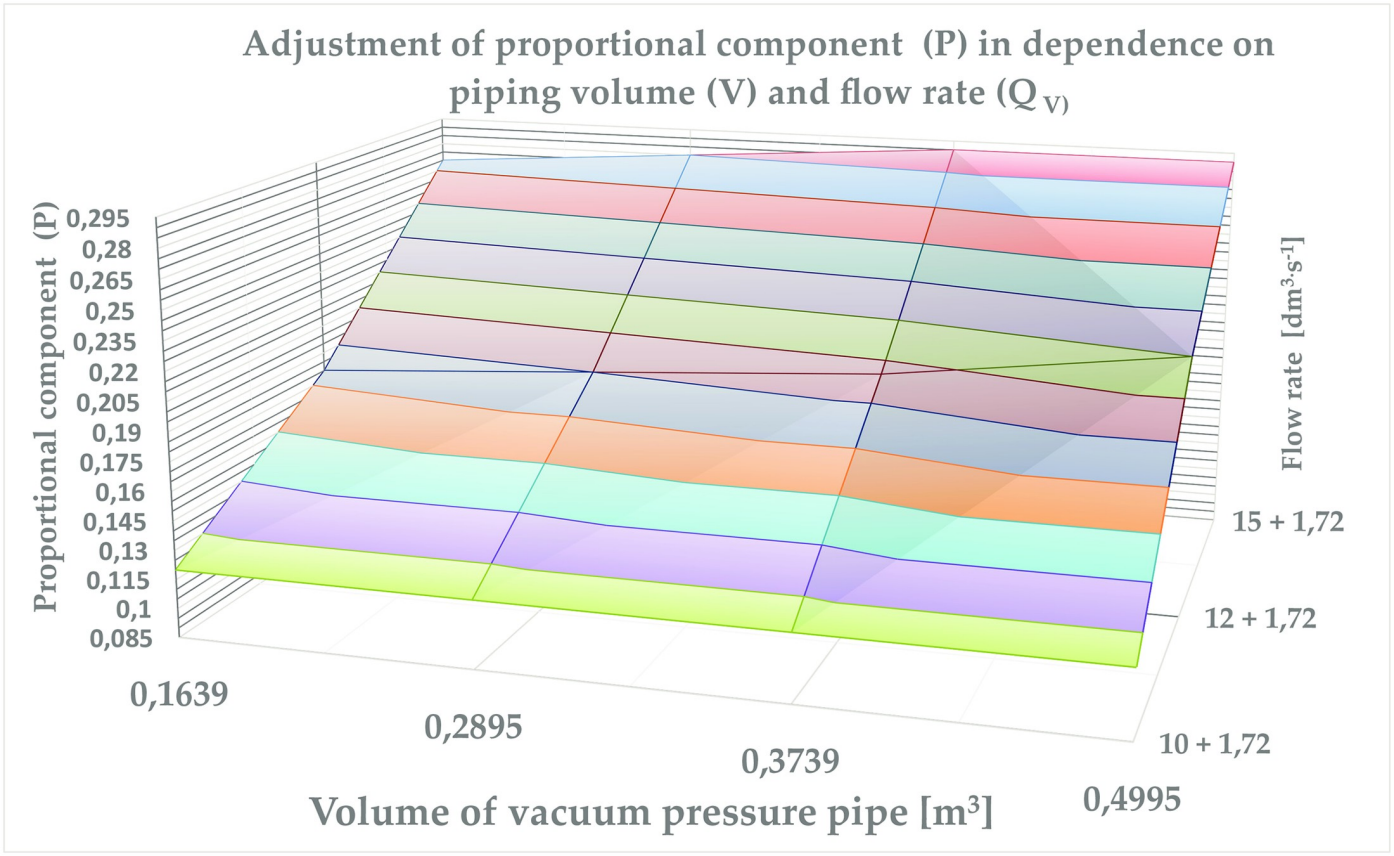
Adjustment of proportional component (P) in dependence on piping volume (V) and flow rate (Q_V_).

The graph shown in Fig 10 and the values in Table 4 describes the change of the proportional component P in relation to the air flow and volume of the vacuum system. It can be stated that increasing the flow rate of air increases the value of the controller parameter (P). The value of the integration component (I) is increased with the increasing volume of the vacuum system while maintaining the amplifying member (P). The result shows the symmetry in the regulation of vacuum values.

**Table 4:** Change of the proportional component P in relation to the air flow.

Volume of Vacuum presure pipe	The actual air flow					
	10 + 1,72 dm^3^ s^−1^		12,5 + 1,72 dm^3^ s^−1^		15 + 1,72 dm^3^ s^−1^	
	P	I	P	I	P	I
0,1639	0,12	0,55	0,18	0,25	0,27	0,3
0,2895	0,12	0,35	0,19	0,25	0,28	0,45
0,3739	0,12	0,45	0,2	0,25	0,29	0,45
0,4995	0,12	0,55	0,22	0,45	0,29	0,25

The statistical evaluation of the variants of the suction and the different volumes proved to be true statements by [36, 37]. The authors state that the system has less amplitude if it has more “capacity”. In this case, the “capacity” is meant by the volume of the vacuum pipe. It can be stated that the correctly set Siemens Sinamics G120P BT will ensure the constant stable vacuum. The control valve in the milking equipment, therefore, can only be used as a safety device if the frequency converter has a fault.

**Fig 11:**
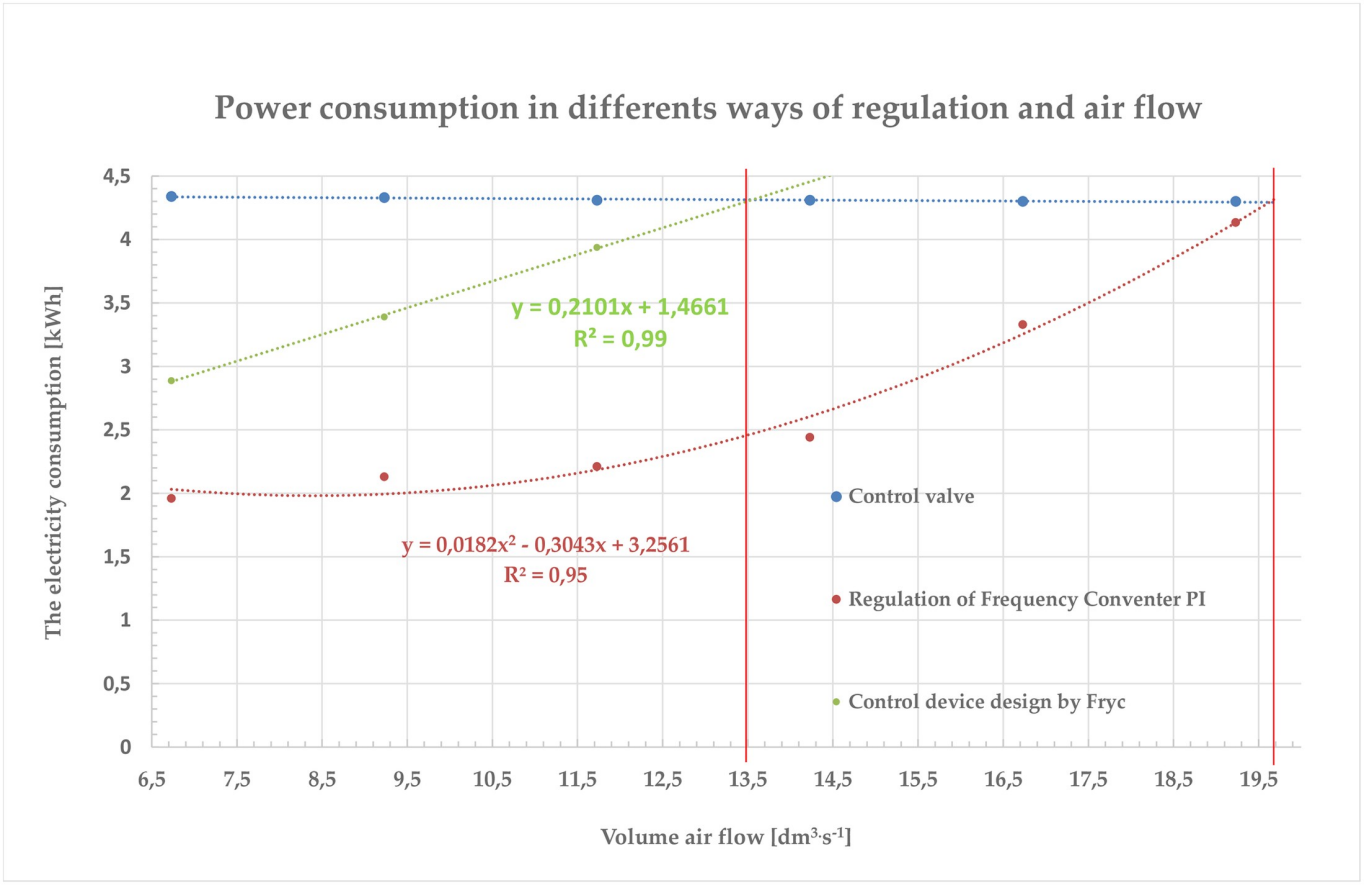
The values of the relative energy savings during the VFD.

In the classical vacuum control with control valve method, the pump’s electric motor still operates at a constant load. This method of regulation is very uneconomical from an energy point of view. Energy losses resulting from improper regulation can account for up to 60% of total power consumption [39]. Statistical evaluation of the vacuum stability data under the control of the Variable Frequency Drive showed that the control valve can be completely disabled. Air consumption by milking units changes rapidly over time. This means that the speed of the pump set must be changed rapidly. The electric motor is almost permanently in the acceleration or braking mode, and thus in the current load condition. Braking is solved by recuperation, and therefore, does not have a negative effect. It is very likely that this regulation will also reduce power consumption [40]. The measurement results of the functional model of the milking unit control by the frequency converter in combination with the device designed by [41] are shown in Fig 11. The values of the relative energy savings during the VFD are positive values in the whole measuring range (from 6.73 dm^3^ s^−1^ to 19.23 dm^3^ s^−1^).

The vacuum pump operates in the range of 7.53 to 15.06 dm^3^ s^−1^. The relative electricity savings are 54.02% to 32.50%. Roşca, R., Cârlescu, P., 2015 published a similar study. The paper reports: Reduction of energy consumption provides technology controlling by VDF, while maintaining the stability of the vacuum [42]. Frequency Converters allows to adjust the amount of air removal from the milking system by changing speed motor of the vacuum pump. Measurements have shown that the application of VDF ensures more accurate stability values of vacuum. The authors statistically compared the regulation using a control valve and VDF [42]. Statistical evaluation shows that there are significant differences between the experimental results.

## Conclusion

The Frequency Converter Siemens SINAMICS G120 with control unit Siemens CU230P-2 is installed on the assembled laboratory milking equipment. The device was identified with the help of first-order transient approximations. Functional mathematical models in Matlab /Simulink 2014a include a PI controller on which different parameters of an amplifier (P) and integration time (I) are chosen. Regulator parameters (PI) are calculated using Ziegler-Nichols methods. The model simulated individual settings that have been validated by real equipment testing VDF. The software Siemens Starter GUi. was used to set the drive parameters. The constructed mathematical model and the real milking system require symmetric regulation.

Statistical methods are used to evaluate vacuum stability. It can be stated that with the increasing flow of the air through the system, the value of the proportional component must be increased in the setting. The controller parameter (integration time I) is increased with the volume of the vacuum pipe as air-flow rate remains the same. Statistical evaluation of the vacuum stability, with the drives Siemens SINAMICS G120 BT showed that it is possible to completely eliminate the control valve. A constant vacuum can be maintained by changing the vacuum pump speed. This procedure complies with the technical standard (ISO 5707: 2007).

The power saving values (on the milking equipment) that control the VFD have been positive throughout the measuring range. The performance of the milking vacuum pump is normally designed from the maximum air consumption of the milking machine at nominal vacuum (50 kPa), and a performance reserve is added to this. This means that the pump is operated between the ranges between 7.53 and 15.06 dm^3^ s^−1^. By using a vacuum pump controlled by a VFD, saves power from 32.50% to 54.02% compared to a control valve. Consequently, the installation of VFD to control the operation of the asynchronous motor and the vacuum itself has proven to be a suitable solution for saving electricity. Their frequent use is encouraged by a relatively low cost price and a short payback period.
